# Patient perspectives on primary care for multimorbidity: An integrative review

**DOI:** 10.1111/hex.13568

**Published:** 2022-09-08

**Authors:** Elaine Moody, Ruth Martin‐Misener, Larry Baxter, Leah Boulos, Fred Burge, Erin Christian, Brian Condran, Adrian MacKenzie, Elizabeth Michael, Tanya Packer, Kylie Peacock, Tara Sampalli, Grace Warner

**Affiliations:** ^1^ School of Nursing Dalhousie University Halifax Nova Scotia Canada; ^2^ Maritime SPOR SUPPORT Unit Halifax Nova Scotia Canada; ^3^ Department of Family Medicine Dalhousie University Halifax Nova Scotia Canada; ^4^ Nova Scotia Health Halifax Nova Scotia Canada; ^5^ Canadian Center for Vaccinology IWK Health Centre Halifax Nova Scotia Canada; ^6^ School of Health Administration Dalhousie University Halifax Nova Scotia Canada; ^7^ School of Occupational Therapy Dalhousie University Halifax Nova Scotia Canada

**Keywords:** chronic disease, integrative review, multimorbidity, primary care

## Abstract

**Introduction:**

Improving healthcare for people with multiple chronic or ongoing conditions is receiving increased attention, particularly due to the growing number of people experiencing multimorbidity (MM) and concerns about the sustainability of the healthcare system. Primary care has been promoted as an important resource for supporting people with MM to live well with their conditions and to prevent unnecessary use of health care services. However, traditional primary care has been criticized for not centring the needs and preferences of people with MM themselves. Our aim was to conduct a review that centred on the perspective of people with MM in multiple ways, including having patient partners co‐lead the design, conduct and reporting of findings, and focusing on literature that reported the perspective of people with MM, irrespective of it being experimental or nonexperimental.

**Methods:**

We searched for published literature in CINAHL with Full Text (EBSCOhost) and MEDLINE All (Ovid). Findings from experimental and nonexperimental studies were integrated into collaboration with patient partners.

**Results:**

Twenty‐nine articles were included in the review. Findings are described in five categories: (1) Care that is tailored to my unique situation; (2) meaningful inclusion in the team; (3) a healthcare team that is ready and able to address my complex needs; (4) supportive relationships and (5) access when and where I need it.

**Conclusion:**

This review supports a reorientation of primary care systems to better reflect the experiences and perspectives of people with MM. This can be accomplished by involving patient partners in the design and evaluation of primary care services and incentivizing collaboration among health and social supports and services for people with MM.

**Patient or Public Contribution:**

Patient partners were involved in the design and conduct of this review, and in the preparation of the manuscript. Their involvement is further elucidated in the manuscript text.

## 1 INTRODUCTION

Multimorbidity (MM)—namely, multiple co‐occurring chronic or long‐term diseases or conditions in one person^1^—affects up to one in four adults worldwide.^2^, ^3^, ^4^ MM is associated with higher costs to healthcare systems^5^, ^6^, ^7^, ^8^ and has been positioned as one of the biggest challenges to sustaining healthcare systems around the world.^9^

Experiencing multiple chronic conditions has consistently been associated with higher mortality, functional decline, and lower quality of life across contexts and measurement approaches.^10^ While these findings demonstrate the impact of MM on a population level, they do little to demonstrate the significant impact of MM on individual lives. MM often contributes to challenges with everyday activities, such as eating, mobilizing, toileting and working.^11^ People with MM often take several medications, may be expected to make significant lifestyle changes, and may require regular self‐monitoring of their health status at home.^12^, ^13^ These characteristics of MM contribute to how people with MM socialize, work, maintain relationships and manage their lives; ultimately, having multiple chronic or on‐going health conditions can impact everyday life in a myriad of ways. The realities of living with MM presents unique challenges that are different than those for someone with a single chronic condition, or an acute health condition. People with MM navigate multiple interacting treatment regimens and their associated adverse effects, have more frequent interactions with healthcare systems and may contend with competing priorities for their time and resources.^4^

Primary healthcare is ideally suited to support people with MM. In principle, primary healthcare recognizes physical, mental and social well‐being as contributing to a person's overall health and extends beyond disease‐centred healthcare to a whole person approach; it also includes health promotion and illness prevention, along with treatment of disease and rehabilitation.^9^ In practice, the term primary healthcare is used to identify a range of services aimed to support the health and well‐being of communities and individuals, which includes services offered by doctors as well as community‐based interventions to address issues, such as housing, infection control and maternal health.^14^, ^15^ This review focuses on primary care (PC)—one service that falls under the umbrella of primary healthcare—which is an approach to supporting individuals' health across the lifespan through connections with family doctors, nurse practitioners or other primary care providers (PCPs) who provide first contact and ongoing access to the healthcare system. Understood in the context of primary healthcare, PCP not only monitor health status and direct medical treatments but they also provide support for self‐management of medical conditions, advocate for changes to social determinants of health and recognize the context of a person's life in their experiences of health and illness.

Increasingly, healthcare leaders and researchers are engaging patients—a term used to describe those with personal experience seeking healthcare, and their family and friend caregivers^16^—in healthcare improvement. Incorporating the perspectives of patients in healthcare design helps make healthcare services more patient‐centred, and ultimately, improve health and organizational outcomes.^16^, ^17^ Patient engagement in healthcare improvement is founded on the idea that patients see healthcare differently than those who have traditionally contributed to healthcare design, such as researchers, policymakers or practitioners.

There is a growing body of literature reporting the perspectives of patients on their PC experiences, and there have been several systematic reviews synthesizing aspects of this literature. For example, there have been reviews examining the perspectives of patients on coordination of PC and oncology care for people with MM,^18^ the perspectives of older people with MM on integrated care,^19^ and the healthcare preferences of older people with MM.^20^ There has not been a review that synthesizes the literature reporting what is important to patients with MM about their PC. While systematic reviews have not traditionally included patient partners, there is growing recognition that having patients involved in literature reviews provide opportunities for interpreting the available literature that provides useful insights into healthcare systems that may otherwise be invisible. Therefore, our aim was to conduct a review of literature reporting on PC for people with MM that foregrounds patients' perspectives in the design, conduct, analysis and reporting of the review, as well as in the content.

## 2 MATERIALS AND METHODS

Integrative reviews are the broadest type of literature synthesis with the purpose of integrating evidence from various types of sources to provide a comprehensive overview of a subject area to inform a specific problem.^21^ This review targets a gap in the literature; while PC has often been understood from the perspective of practitioners, researchers and policymakers, the perspectives of people with MM about what they feel is important about PC have not been synthesized. The current review was conducted to contribute to the evidence supporting quality improvement initiatives in PC to better reflect the circumstances and needs of people with MM. There are no PRISMA reporting guidelines for integrative reviews; however, we followed the 2020 PRISMA guidelines for reporting the literature selection process.^22^ The question guiding the review was: What do people with MM want their PC to look like?

With support from a librarian trained in systematic and integrative review methods (Boulos), we developed a search strategy using the key concepts: MM, PC and patient perspectives. A series of exploratory scoping searches conducted in 2017 to support the design of our project revealed a broad body of literature related to these key concepts. We determined that it would be difficult to capture the breadth and depth of the literature in a single systematic or scoping review. Therefore, we used scoping searches to develop a search strategy for this integrative review. First, we identified a preliminary set of literature, then harvested the subject terms and keywords from this set of literature and finally used those terms and keywords to develop a targeted search appropriate for this integrative review. The search was run in CINAHL with Full Text (EBSCOhost) and MEDLINE All (Ovid) on 11 February 2020 (see Appendix 1 for comprehensive search strategy). These databases were chosen because of their breadth of coverage of biomedical and allied health literature. No search filters, date limits or language limits were applied to the search, but only English resources conducted since 2005 were included during the screening process. We sought theoretical as well as empirical work and included articles from qualitative, quantitative and mixed method research traditions. The sources identified in the comprehensive search were uploaded to Covidence (Veritas Health Innovation, Melbourne, Australia) for deduplication and screening. Two reviewers independently screened all titles and abstracts for meeting the inclusion criteria. Studies that progressed to full‐text screening were uploaded to Covidence and screened independently by two reviewers. Any disagreements at the title and abstract or full‐text stages were resolved by discussion or by a third reviewer. Articles were included if they (1) focused on a population with two or more chronic or on‐going health issues, (2) presented results from the perspective of people with MM and (3) discussed PC services or providers. Inclusion and exclusion can be found in Table 1. Once the final articles were selected, we reviewed the reference list of each to identify other relevant literature. No new sources were added at this stage.

**Table 1:** Inclusion and exclusion criteria

Inclusion criteria	Exclusion criteria
Provides a patient perspective (i.e., primary source of data is patients).	Written in languages other than English.
People with multimorbidity (or multiple chronic conditions, etc.) including two or more conditions, which may include mental or physical conditions, and may also include obesity or addiction.	Published before 2005.
Primary care (i.e., services or providers), the point of access to health care, including family doctors, nurse practitioners and family practice nurses.	Protocols, conference abstracts, reviews.
Research articles (qualitative, quantitative or mixed methods) or theoretical papers.	Articles that focus on an intervention that is not the services or providers of primary care (e.g., perspectives on electronic medical records [EMRs]).

Evaluating and comparing the quality of studies from diverse methodological traditions in the context of integrative reviews can be complex.^21^ For a review such as this, which includes studies using various research methods, it is appropriate to evaluate methodological quality in a manner that is specific to the method used. We conducted quality appraisal using the Critical Appraisal Skills Programme Qualitative Research Checklist^23^ for qualitative studies; the AXIS tool^24^ for cross‐sectional and observational studies and the JBI quality appraisal tool for cohort studies.^25^ We included all studies that met the inclusion criteria regardless of methodological quality but used the quality appraisal to examine outlier cases in the findings.^21^

This review was part of a larger study examining patient perspectives on team‐based and patient‐centred PC, which involved ongoing engagement of patient partners (Baxter and Peacock). Not only did the patient partners have lived experience of MM but they had also been involved in research and advocacy for issues related to MM over time, including collaborations with several other team members. Baxter and Peacock were involved in conceptualizing the objectives of the review, designing the search strategy, screening the literature and interpreting the results. The patient partners were significantly involved in identifying key elements of the review that reflected their experiences and priorities, and they provided insights into how the literature could be interpreted through the lens of a person with MM. To help accomplish meaningful engagement, the review team met weekly over the course of the review, as part of on‐going team meetings for multiple projects over several years.

**Figure 1:**
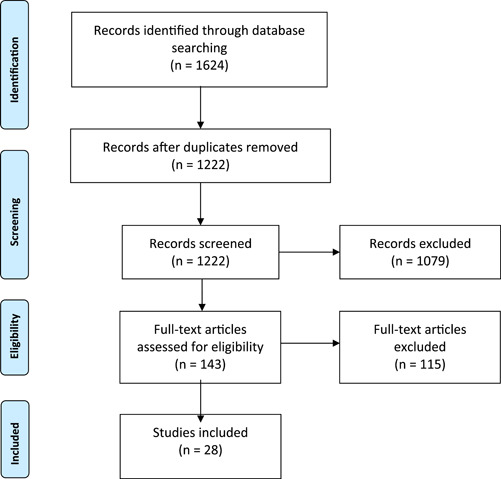
PRISMA diagram.

Data analysis progressed through five phases: data reduction, data display, data comparison, conclusion drawing, and verification.^21^ For data reduction, we developed a classification system comprising focused questions that were developed from our study objectives and that we applied to each source (see Table 2 for sample questions). A table was designed to display the results of data reduction and was used to facilitate data comparison. We then used methods similar to thematic analysis to look for commonalities, contrasts and other relationships within the data table through an iterative process.^26^ The result of the data comparison phase was a narrative of the findings. In the final verification process, both the narrative and data tables were reviewed by our patient partners to develop further analytical questions to apply to the original sources and expand interpretations. For example, they shared that from a patient perspective, the healthcare team included family members, PCP and medical specialists. They also identified the language to translate the research findings into concepts that reflect the lived experience of people with MM. Thus, the results are presented, as much as possible, with the patient perspective at the fore Table 3, Figure 1.

**Table 2:** Sample questions from the classification system used for data reduction

What were the challenges or barriers to primary care (PC) identified by patients?
What PC supports or resources did patients identify?
What did patients want in relation to the form, function and attributes of PC?
What factors did patients identify as contributing to successful encounters in PC?

## 3 RESULTS

### 3.1 Included articles

A total of 1624 sources were identified. Of these, 402 were duplicates. The titles and abstracts of 1222 sources were screened and 1079 were not relevant. The full‐text screening phase included 143 sources of which 115 were excluded. See Figure 1 for the PRISMA diagram. A total of 28 sources were included in the review. A summary of the included sources can be found in Table 3. Of the included studies, 11 were from the United States,^27^, ^28^, ^29^, ^30^, ^31^, ^32^, ^33^, ^34^, ^35^, ^36^, ^37^ four were from the United Kingdom,^38^, ^39^, ^40^, ^41^ three were from Canada,^42^, ^43^, ^44^ three were from Denmark,^45^, ^46^, ^47^ two from New Zealand,^48^, ^49^ one each from France,^50^ Germany,^51^ Norway,^52^ Spain^53^ and the Netherlands.^54^ Most of the included articles used qualitative methods including semi‐structured interviews^32^, ^34^, ^37^, ^38^, ^40^, ^42^, ^44^, ^45^, ^46^, ^52^ (*N* = 11), focus groups^33^, ^36^, ^47^ (*N* = 3), a discrete choice experiment^50^ (*N* = 1), ethnographic methods^41^ (*N* = 1) and a combination of interviews and focus groups^48^ (*N* = 1). Nine included studies used cross‐sectional surveys to collect data,^27^, ^29^, ^30^, ^35^, ^39^, ^49^, ^51^, ^53^, ^54^ one used an observational design^28^ and one used retrospective cohort analysis.^31^

**Table 3:** Summary of included articles

First author	Year	Country	Design	Population	Purpose
Adeniji	2015	UK	Cross‐sectional questionnaire	People experiencing multimorbidity, with the presence of two of the following conditions: COPD, CAD, diabetes, OA and depression.	‘Explore the experience of “hassles” among patients with multimorbidity in primary care in the UK’.
Arreskov	2018	Denmark	Semi‐structured interviews	Participants were diagnosed with nonmetastatic cancer with completed primary treatment and comorbidities of diabetes, COPD and/or CVD.	‘Explore patients' experiences of living with a cancer diagnosis and pre‐existing comorbid chronic conditions and the possible effects on everyday living and management of the comorbidities’.
Bayliss	2008	USA	Semi‐structured interviews	65 And older, at minimum have the conditions of diabetes, OA and depression.	‘Our intent was to explore patient perspectives on components of “best” processes of care for persons with multiple morbidities to inform the development of future interventions to improve care’.
Benzer	2019	USA	Observational study using survey and administrative data	Veterans with at least two inpatient or outpatient encounters for diabetes and two encounters with cardiovascular and mental health comorbidities.	‘To empirically test patient and disease characteristics that may influence patient‐experienced coordinated care’.
Berntsen	2018	Norway	Semi‐structured interviews	Participants with a wide range of long‐term health challenges including cancer.	‘Explore how the PC‐IC process ideal might be useful as a guide to capture iPP quality, and… operationalize… into a quality of care framework’.
Birke	2019	Denmark	Focus groups	Patients from the clinic with two of the following conditions: diabetes, heart disease and lung disease.	Assess the feasibility of the model in practice for further RCT evaluation.
Corser	2011	USA	Semi‐structured interviews and retrospective chart audits	Participants had a diagnosis of two of the following: diabetes, chronic pulmonary disease, congestive heart failure, CAD, OA/musculoskeletal disorder and/or ongoing cancer.	Explore perceived needs of adults with MM regarding self‐management and relationships with PCP.
Cowie	2009	UK	Semi‐structured interviews	Participants were being managed for one of seven conditions: arthritis, CAD, stroke, hypercholesterolaemia, hypertension, diabetes mellitus or COPD.	(1) Evaluation if patients' experiences are consistent with a common conceptual model, and (2) exploration of the potential influence, of condition or variations in the organization/delivery of care on patients' experiences.
Ehman	2017	USA	Cross‐sectional survey	Patients received surveys if they had an assigned PCP, were 18+, able to fill out the survey in English and were part of the clinic before team care.	Determine preference for continuity and access to care b/t healthy patients and those with MM.
Fortin	2010	Canada	Semi‐structured interviews	Patients with 5+ chronic conditions from one of the following clinics: FMG, CLSC and FMU.	Explore perceptions and expectations of patients with MM in regard to nurses' involvement in primary care practices.
Gill	2014	Canada	Semi‐structured interviews	65 Years of age or older, diagnosed with two or more chronic conditions, had an informal caregiver who participated in the patient's healthcare, spoke English as a first language and was able to provide informed consent.	Understand health system experience to demonstrate where improvements are most needed to manage multimorbidity.
Goldberg	2019	USA	Repeated cross‐sectional telephone survey	Adults who were covered by CareFirst insurance, whose primary care provider participated in the CareFirst PCMH programme, and had multiple chronic conditions.	‘Examine the care experiences of patients with primary care providers in a payer‐based PCMH programme at two points in time’.
Hays	2017	UK	Ethnographic methods	65+, Two or more active long‐term conditions.	‘…to identify and describe threats to patients safety in primary care among older people with multimorbidity, to provide a better understanding of how these are experienced and to inform the development of interventions to reduce risks to patient safety’.
Janke	2016	USA	Semi‐structured interviews	Participants had: (1) BMI > 25; (2) weekly pain intensity >4/10 for prior 3 months; (3) diagnosis of persistent pain.	Examine perceptions of patients with obesity and chronic pain regarding PCP care management.
Kerrissey	2017	USA	Cross‐sectional survey	Older adults with two or more chronic conditions.	Report on the empirical relationship between integrated care domains (6) and medical group structural characteristics.
Knowles	2015	UK	Semi‐structured interviews	Participants with a diagnosis of CHD and/or diabetes.	Inform the development and implementation of future collaborative care models.
Kristensen	2018	Denmark	Semi‐structured interviews	Participants had diabetes and one or more other chronic condition and impaired self‐care ability.	Investigate the experiences of disease and self‐care ability, and the patient's view of the GP's role in self‐care.
Krucien	2015	France	Discrete choice experiment	OSAS and an additional chronic condition.	Identify the preferences of patients with MM within the Chronic Care Model.
Kuipers	2019	the Netherlands	Cross‐sectional survey	Participants had two or more chronic conditions.	Examine level of PCC for MM patients in PC, including relationships b/t PCC, cocreation of care, care satisfaction and well‐being of patients.
Matthias	2010	USA	Focus groups	Participants had chronic pain and depression.	Patient perceptions of relationship with NCM and PCP and influence on pain self‐management.
McKinlay	2015	New Zealand	Interviews and focus groups	Participants had three or more long‐term conditions.	Views of diverse MM patients and available healthcare.
Millar	2018	New Zealand	Cross‐sectional survey	Patients with two or more long‐term conditions.	‘Understand the experiences of people with MM in the NZ health care system’.
Mortsiefer	2017	Germany	Secondary analysis of survey	Participants had three or more long‐term conditions.	Assess how patients with MM evaluate GP and identify factors for higher patient satisfaction.
Noel	2005	USA	Focus groups	Patients with two or more chronic conditions.	Explore problems of patients with MM, communication with PCP, self‐management and monitoring/follow‐up preferences.
Parchman	2005	USA	Cross‐sectional survey	More than one chronic illness.	‘Examine the relationship between attributes of primary care and health care system hassles among veterans with 1+ chronic illnesses’.
Rincon‐Gomez	2011	Spain	Survey	Assigned to study primary care centres and meeting polypathological criteria.	Examine the perceived quality of care for patients with polypathology.
Roberge	2016	Canada	Semi‐structured interviews	18+, presence of chronic disease, depression/anxiety in past 2 years (diagnosed), have a family physician in the study clinic.	Study of the perceptions of clinicians and patients regarding services for depression/anxiety for those with chronic conditions in primary care.
Salzberg	2016	USA	Retrospective cohort analysis	18+ and 3+ chronic conditions.	Compare health experiences of adults with MM and limited function to adults with MM with no functional limitations.

Abbreviations: CAD, coronary artery disease; COPD, chronic obstructive pulmonary disease; CVD, cardiovascular disease; GP, general practitioner; MM, multimorbidity; OA, osteoarthritis; OSAS, obstructive sleep apnoea syndrome; PCP, primary care provider; UK, United Kingdom; USA, United States of America; NCM, nurse case managers; PCMH, patient centered medical home. RCT, randomized controlled trial.

### 3.2 Main findings

In reviewing the literature reporting the perspectives of people with MM on PC experiences, we found five characteristics of PC that were particularly important to people with MM (see Table 4 for an overview of the findings). First, people with MM wanted a PC that was tailored to their unique situation. This reflects the complexity of MM and how individual contexts impact how care should be designed, and how decisions should be made reflecting collaboration between patients and providers. Second, people with MM wanted to be meaningfully included in their healthcare team. Meaningful inclusion means that they are listened to and appreciated for their expertize by all team members. Third, people with MM identified the importance of having a healthcare team that was ready and able to address their complex needs. While people with MM want to be active participants in their healthcare team, they depend on a high‐functioning healthcare team that provides coordinated and expert care. Fourth, people with MM relied on mutually respectful relationships with healthcare providers to ensure treatments and recommendations fit their life, and that they could access information about the health system when and how they needed it. Fifth, people with MM valued having access to care when and where they needed it. This means that timely access to appointments for urgent health concerns, alternatives to in‐person clinic appointments and timely access to other healthcare resources such as specialists are important to people with MM. In the following presentation of the five characteristics, there are many intersections and connections that signify that the boundaries of each characteristic are somewhat artificial and that ultimately, they come together to describe what patients think it might be like to have PC that reflects their needs. Moreover, the results are intended to provide a different way of looking at PC that comes closer to how people with MM themselves see the role of PC in their lives, rather than providing a comprehensive description of ideal PC services.

**Table 4:** Overview of findings

Care that is tailored to my unique situation.
Meaningful inclusion in the team.
A healthcare team that is prepared and able to address my complex needs.
Supportive relationships.
Access when and where I need it.

### 3.3 Care that is tailored to my unique situation

In the context of MM, it is particularly urgent to adapt care practices, treatment options and lifestyle recommendations to each person's particular situation because of the complexity that often accompanies MM. Patients with MM valued having comprehensive, individualized care that reflected the complexity of their particular situations, including their social, medical and environmental circumstances, to ensure that treatments and care recommendations were feasible and realistic.

The literature demonstrated that, for people with MM, it was particularly important to have information about the interaction of their multiple conditions, and not solely on each condition separately.^28^, ^32^ Benzer et al.^28^ found that those with more health conditions experienced higher rates of fragmentation of information and less communication about treatments. This becomes increasingly important when people with MM are accessing care across settings; the authors found that having information that was consistent and continuous across settings and providers was important to prevent conflicting advice or gaps in the information. People with MM need to have their multiple conditions addressed together rather than as single conditions in isolation from each other.

Cowie et al.^38^ described how it was important to people with MM that they were able to discuss several health issues with their PCP at a given time. People with MM wanted support with lifestyle changes and other strategies that supported their comprehensive health and wellness, such as illness prevention,^51^ support for self‐management of their MM,^29^, ^48^ and support with managing multiple medications.^29^ Such support required PCPs to consider the interaction of their conditions and treatments, as well as to prioritize aspects of their care. Arreskov et al.^45^ reported on the perception of PC patients with cancer and other chronic conditions and noted that they wanted their PCP to recognize and prioritize their chronic disease management when it was important to them, rather than always focusing on their cancer management to the exclusion of other aspects of MM.

People with MM wanted PC to be focused on their comprehensive, holistic health, and not solely on their physical health conditions. Several studies noted that people with MM wanted support for their mental and emotional well‐being, along with their physical health.^33^, ^37^, ^40^, ^44^, ^54^ Roberge et al.^44^ noted that patients with MM felt stigmatized when they had mental health conditions. Also in relation to mental health care, team‐based PC approaches were discussed in several studies. Knowles et al.^40^ reported that patients accessing PC through a collaborative team with expertize in mental and physical health needs found that the team approach enabled access to both physical and mental health supports, rather than patients having their mental health needs to be addressed separately and independently. Roberge et al.^44^ found that patients with mental and physical health conditions liked to have access to various types of providers, including physicians, nurses and psychologists. Matthias et al.^33^ found that patients with chronic pain appreciated emotional support from nurse case managers providing pain care. Attention to mental and emotional needs was important to people with MM.

Part of tailoring care to the person's particular situation was ensuring that information about their health and treatments were in accessible formats.^35^, ^39^, ^54^ For example, people with MM wanted instructions for lifestyle changes, treatment plans and future services to be personalized and written down so that they were clear and there was little room for ambiguity.^28^, ^34^ They also needed health information in their first language, not necessarily the language spoken in the healthcare environment,^48^ further supporting the need for information that is created for the particular person with MM.

### 3.4 Meaningful inclusion in the team

To tailor care to the person's situation, people with MM need to be meaningfully included in healthcare teams and respected for their expertize. This was often described in the literature as wanting to be heard and understood, along with being well‐informed.^33^, ^34^, ^37^, ^47^, ^49^ Patients with MM wanted to have direct and consistent communication between themselves and their PCP.^31^, ^36^, ^41^, ^43^, ^44^

Part of being valued as a member of the healthcare team was feeling like they were heard, and that providers actively listened to their concerns and responded appropriately.^33^, ^41^, ^48^, ^51^ Janke et al.^32^ noted that the ways providers communicated with patients were important to how patients perceived their overall care and affected their perception of how invested their providers were in their care. Noel et al.^36^ reported that at times patients had challenges getting their provider to take their concerns seriously. Matthias et al.^33^ found that patients attributed challenges in their treatment plans, such as being over‐ or under‐prescribed, as resulting from providers not listening to their perspective. Birke et al.^47^ found that people with MM wanted their knowledge and priorities to be taken into consideration in the planning process; however, they also noted that such information was not collected in any systematic way. In a study by Millar et al.,^49^ patients found it particularly challenging when their concerns were ignored; they wanted to have their opinions respected.

Another aspect of being part of the team was being informed about important elements of their care. Adeniji et al.^39^ studied PC hassles—situations that created problems or inconveniences—as reported by people with MM and found the ‘biggest hassles’ were a lack of information about health conditions and treatment options. In a study by Kristensen et al.,^46^ people with MM said they wanted their providers to give them a clear rationale for treatment decisions and referrals.

### 3.5 A healthcare team that is ready and able to address my complex needs

While it was important to people with MM to be respected as experts in their healthcare team, they also wanted their PC team to take responsibility for ensuring their healthcare needs were addressed. While a person with MM may recognize gaps in their care, they do not necessarily have the knowledge, access to the health system, skills or confidence to address the gaps, particularly while contending with multiple chronic health conditions. People with MM relied on the expertize of PCPs to ensure there were no gaps in their care. Some people with MM reported that poorly coordinated care and communication breakdowns put them at risk of poor health.^37^, ^43^, ^47^ Birke et al.^47^ reported that people with MM felt forced to become more involved in their care than they would have preferred, to ensure that treatments or other aspects of their care were not missed. Gill et al.^43^ reported that people with MM felt poorly coordinated care contributed to not being able to access medication, having to wait for tests or procedures and not getting test results in a timely manner. Similarly, Janke et al.^32^ found that people with MM wanted their PCP to initiate teaching about how to maintain motivation, rather than having to research such topics themselves.

From the perspective of people with MM, it is important to have a cohesive healthcare team. While healthcare systems are often compartmentalized by care setting, disease or body system affected, such compartmentalization interferes with addressing the complex challenges in the lives of people with MM. Several studies noted the importance of the PC team connecting with other professionals involved in their care, such as medical specialists.^36^, ^39^, ^47^, ^50^ To ensure their healthcare was integrated with their unique situation, it was important for people with MM that their family members and community supports were included as healthcare team members.^53^, ^54^

From the perspective of people with MM, it was essential for their healthcare team to have excellent communication to effectively address their complex needs.^35^, ^36^, ^37^, ^38^, ^39^, ^41^, ^43^, ^47^, ^49^, ^50^ This included communication within the PC team itself, as well as with other community and healthcare resources.^41^, ^42^, ^43^ A study by Goldberg et al.^27^ examined the role of care plans in PC and found that people with MM found them to support their care, and noted that incomplete care plans were a barrier to good care.

### 3.6 Supportive relationships

People with MM valued having relationships with care providers that were sustained over time and exemplify mutual respect. The relationships that were most valued were those that reflected a genuine concern, the memory of the person's situation and care needs and being responsive to the individual's concerns.^36^, ^46^, ^49^, ^53^ People with MM found it difficult when their providers forgot aspects of their care, such as medications or specific treatments.^34^, ^49^

The literature reporting on relationships between people with MM and PCP often draws on the concept of continuity, which is recognized as a particularly beneficial and desired aspect of PC. There are various theoretical descriptions of continuity, some of which further describe three types of continuity: relational, informational and managerial continuity.^55^ Relational continuity generally reflects having the same provider over time. It was not a surprise, therefore, to find that many of the studies in this review reported that people with MM wanted to see the same providers over time.^30^, ^38^, ^41^, ^42^, ^46^ Fortin et al.^42^ found that patients with MM had higher expectations for continuity when nurses worked with physicians in PC practices; they expected to see the same nurse over time. A study by Ehman et al.^30^ compared the perspectives of people with MM and those with single conditions on whether they preferred waiting to see their usual provider or being able to see a different provider more quickly. The results demonstrated that all patients, including those with MM, preferred quick access to any PCP for acute conditions rather than waiting to see their usual care provider. However, they also found that those with MM were willing to wait longer than those without MM to see their usual provider for chronic issues. This suggests that relationship continuity may be particularly important to people with MM.

Several studies found that patients with MM liked to have a ‘point person’, such as a care manager or care coordinator—someone they could reach out to with questions, who would have a sense of their history and current situation.^33^, ^34^, ^41^, ^43^, ^46^ This has been referred to as managerial continuity.^55^ Bayliss et al.^34^ described the value of a care manager/coordinator to people with MM as someone who provided continuity, served as a liaison between healthcare team members and helped prioritize health issues. In a study by Kristensen et al.,^46^ people with MM viewed their PC physicians as health consultants, someone who understood their situation and could provide information to better manage their health conditions. Matthias et al.^33^ found that patients preferred having a nurse case manager as their primary contact rather than a family doctor. The authors suggested this was because the nurse case manager and patient developed a stronger and more beneficial relationship.

### 3.7 Access when and where I need it

Central to patients' experiences of PC was the process of accessing healthcare expertize when and how they needed it. Traditionally, PC has been organized around in‐person appointments at healthcare clinics, where patients are scheduled to see their healthcare provider at a particular time and for a set period. Several of the included papers noted that people with MM found this structure difficult. For people with MM, who access healthcare more often than those with single conditions, having to wait for access can lead to neglect of other aspects of their care.

People with MM had trouble when there were delays in accessing healthcare, whether from their PC team or from other healthcare providers.^27^, ^33^, ^37^, ^41^, ^43^, ^44^ One recurring issue in the literature was having to wait for appointments; people with MM reported having to wait longer than they needed or wanted to see their PCP.^35^, ^43^, ^49^, ^51^, ^52^, ^53^ The literature also noted people with MM experienced unacceptable delays in accessing other healthcare services that were coordinated by their PC teams, such as specialist services and consultations.^31^, ^34^, ^36^, ^38^, ^39^, ^49^ People with MM also experienced challenges when they had to spend a prolonged period waiting to see their PCP in the clinic waiting room.^53^

For people with MM, it is often a challenge to discuss all of their issues in the timeframe of standard appointments. Across several studies, people with MM noted a preference for longer appointment times so they could address several concerns at a time, rather than make multiple appointments to address their concerns.^33^, ^48^, ^52^ Birke et al.^47^ found that people with MM reported better coordination of care when they had more time with their general practitioner, which ultimately improved individualized care. In a study examining specifically how the involvement of nurses in PC teams was experienced by people with MM, Fortin et al.^42^ found that patients expected better accessibility (i.e., more timely access) when nurses were part of a PC clinic. The authors suggested that the expectation for increased accessibility was, at least in part, due to patients assuming nurses could facilitate access to doctors in situations where the patient needed to be seen urgently.

The literature suggests that people with MM would like convenient access to their healthcare providers that aligns with their needs.^37^, ^41^ Many of the sources reported that people with MM wanted a way to connect with their PCP on short notice, such as making same‐day appointments,^48^, ^51^ urgent appointments^36^ and access to providers between scheduled appointments.^35^ While the review does not reflect patient perspectives on the move to virtual care due to the COVID‐19 pandemic, it does show that people with MM supported alternatives to the traditional structure of PC, including telephone conversations,^36^ access to care on evenings, weekends and holidays^27^ and email access to providers.^34^, ^36^

## 4 DISCUSSION

Through conducting this review, we found that it was important for people with MM to be respected as part of the healthcare team and to have their voices heard, to ensure that their care was specific to their complex needs. At the same time, it was also important to them to have a PC team they could rely on to coordinate their care knowledgeably and skillfully. People living with MM highlight the importance of individualized approaches to care, including considering multiple conditions together, along with other social and emotional elements of each person's life, in the planning and delivery of their care. Ultimately, for people with MM, these characteristics of PC were more than a preference; they saw them as essential to quality care, and without them, they felt at risk of negative outcomes.^37^, ^43^, ^47^ Due to the complex and often unique nature of their conditions, gaps in services can at worst be life‐threatening and may contribute to poor quality of life.

### 4.1 Strengths and limitations

Integrative reviews provide an exceptional opportunity to integrate research using experimental and nonexperimental methods and to draw on the experience of patient partners to integrate findings.^21^ For our review, which focused on studies that described the perspectives of people with MM, it was particularly valuable to have patient partners with lived experience of MM to interpret the findings. This engagement was not superficial; it provided instrumental interpretations to see the body of literature and PC in a new way. While this review provides important insights into how people with MM view PC, it also has limitations. In general, integrative reviews are limited in their ability to support a meta‐analysis of research findings to make generalizable recommendations. Our search strategy included a comprehensive search of two digital databases using search terms and keywords identified through relevant literature and an analysis of reference lists of the included sources. It is possible that this strategy may not have retrieved all literature on the topic. Additionally, as the search was conducted in 2020, the review is limited to studies published to that point.

### 4.2 Implications for healthcare design and delivery

In many ways, the findings from this review focused on the perspectives of people with MM and reflect a wider body of literature demonstrating the value of person‐centred, comprehensive and integrated PC as the best way to support people with MM. Case management is a mainstay of new models of PC for people with MM.^56^ The importance of continuity in relation to PCP is well supported, particularly for caring for people with MM.^55^ PC models developed over the past two decades have often explicitly focused on patient‐centred care and are anticipated to continue to do so over the next few decades.^57^, ^58^ However, there continue to be challenges in addressing the complex needs of people with MM. There is little research evidence about how to address MM,^12^ and few clinical guidance documents are available to support PCP working with this population.^59^ This review provides some direction about areas where small and large changes can be implemented to make PC more patient‐centred.

One area where change has been swift and likely to result in long‐term changes to how PC is provided to people with MM is the use of e‐health strategies, which have been accelerated by necessity due to the COVID‐19 pandemic. The current review found that people with MM appreciated having multiple methods of contacting their providers, particularly in relation to issues where patients wanted to speak to a healthcare provider urgently, such as same‐day or next‐day appointments. Virtual care may also provide opportunities for access to PC outside regular work hours, such as weekends and evenings, which would also support more flexible work hours for providers.

While technology will likely play a significant role in the evolution of PC for people with MM, it is clear from this review that relationships continue to be a mainstay of PC. We found that the central issues for people with MM were to have time with providers to adequately address their issues and that conversations were focused on the person's goals, preferences and specific context. Indeed, when PCP are challenged by multiple guidelines in relation to the care of people with MM, they often fall back on person‐centred and individualized strategies to make recommendations to support their health and minimize risk.^59^ There has been a general movement away from relying on professional judgement to having explicit guidelines to provide direction to professionals.^60^ Ultimately, however, it may not be useful to hold individual PCP exclusively responsible to ensure that the needs of people with MM are met. Lingard^61^ suggests we might do better to focus on collective competence—the effective working of a group—rather than individual competence. With the growing use of collaborative teams in providing PC, particularly for people with MM, it may be useful to consider how a collective competence approach that includes patients could be translated to PC teams. Such an approach would require system‐level support, such as integrated information systems across healthcare settings. This review demonstrates that one of the most significant challenges for people with MM is fragmented care. If there is a way to draw the focus away from individuals, whether PCP, specialists or others, towards the healthcare system, as a whole, being accountable for patient care, it might reorient the system to addressing the needs of the person, rather than relying on each individual provider to do their own job well without any system for ensuring collaboration.

While there are likely small changes that can be made to continue to improve the PC of people with MM, such as addressing the structure of appointment making and continuing to build on strategies that identify a point person, ultimately it is necessary to make significant changes to how PC is organized and funded to make the changes necessary to meet the challenges of MM. It will take courageous action by politicians and healthcare leaders to make changes in the fundamental healthcare structure. Stott and Young^62^ suggest that current systems reward costly tertiary and secondary treatments, and create barriers to strategies that might more effectively address patient needs—namely that they have someone to turn to, have support with lifestyle changes and someone knowledgeable and skilled who genuinely cares about them. With this in mind, it would be necessary to examine how we evaluate the effectiveness of PC and the outcomes measured to demonstrate changes in PC. In discussing integrated care models, Hughes et al.^56^ suggest it might be important to evaluate models of care based on their meaning to patients, rather than their ability to predict population‐level health outcomes. This review highlights how current approaches risk patients' needs being overlooked or unnoticed by anyone in the healthcare system. It is therefore important to find better ways to involve patients with MM in the design and evaluation of new approaches to the care of patients with MM in PC.

## AUTHOR CONTRIBUTIONS

**Elaine Moody**: Conceptualization (equal); formal analysis (lead); writing – original draft preparation (lead). **Ruth Martin‐Misener**: Conceptualization (lead); formal analysis (equal); funding acquisition (lead); writing – original draft preparation (equal). **Larry Baxter**: Conceptualization (equal); formal analysis (lead); validation (lead). **Leah Boulos**: Methodology (lead); writing – review and editing (equal). **Fred Burge**: Conceptualization (equal); formal analysis (equal); writing – review and editing (equal). **Erin Christian**: Conceptualization (equal); writing – review and editing (equal). **Brian Condran**: Analysis (supporting); project administration (lead). **Adrian MacKenzie**: Conceptualization (equal); formal analysis (equal); writing – original draft preparation (equal). **Elizabeth Michael**: Conceptualization (equal); writing – review and editing (equal). Tanya Packer: Conceptualization (equal); formal analysis (equal); writing – review and editing (equal). **Kylie Peacock**: Conceptualization (equal); formal analysis (lead); validation (lead); writing – review and editing (equal). **Tara Sampalli**: Conceptualization (equal); formal analysis (equal); funding acquisition (lead). **Grace Warner**: Conceptualization (equal); formal analysis (equal); supervision (lead); writing – review and editing (equal).

## CONFLICT OF INTEREST

The authors declare no conflicts of interest.

## Data Availability

Data sharing is not applicable to this article as no datasets were generated or analysed during the current study.

## APPENDIX 1 SEARCH STRATEGIES

### MEDLINE(R) All (Ovid)

**Table:**

1	exp *Primary Care/
2	(primary care or primary care or primary care).ti,ab.
3	(general practi* or family doctor? or family physician?).ti,ab.
4	(“continuity of care” or “continuity of patient care” or “continuum of care” or “continuum of patient care”).ti,ab.
5	(patient centred or patient centred or patient focused).ti,ab.
6	patient navigat*.ti,ab.
7	medical home?.ti,ab.
8	or/1‐7
9	exp *Comorbidity/
10	Multiple Chronic Conditions/
11	(comorbid* or co morbid*).ti,ab.
12	(multimorbid* or multi morbid*).ti,ab.
13	multiple morbid*.ti,ab.
14	(multidisease? or multi disease?).ti,ab.
15	((concurrent or cooccur* or co occur* or multi or multiple or “more than one”) adj2 (condition? or disease? or disorder? or illness* or syndrome?)).ti,ab.
16	or/9‐15
17	exp *Patient Satisfaction/
18	(patient* adj2 (desire? or experience? or need? or opinion? or prefer* or priorit* or satisf* or value?)).ti,ab.
19	(patient* adj2 (barrier? or challenge? or enable* or facilitat* or obstacle?)).ti,ab.
20	or/17‐19
21	8 and 16 and 20

### CINAHL with Full Text (EBSCOHost)

**Table:**

1	(MM “Primary Care”)
2	TI(“primary care” OR “primary care” OR “primary care”) OR AB(“primary care” OR “primary care” OR “primary care”)
3	TI(“general practi*” OR “family doctor#” OR “family physician#”) OR AB(“general practi*” OR “family doctor#” OR “family physician#”)
4	TI(“continuity of care” OR “continuity of patient care” OR “continuum of care” OR “continuum of patient care”) OR AB(“continuity of care” OR “continuity of patient care” OR “continuum of care” OR “continuum of patient care”)
5	TI(“patient centred” OR “patient centred” OR “patient focused”) OR AB(“patient centred” OR “patient centred” OR “patient focused”)
6	TI(“patient navigat*”) OR AB(“patient navigat*”)
7	TI(“medical home#”) OR AB(“medical home#”)
8	S1 OR S2 OR S3 OR S4 OR S5 OR S6 OR S7
9	(MM “Comorbidity”)
10	TI(comorbid* OR “co morbid*”) OR AB(comorbid* OR “co morbid*”)
11	TI(multimorbid* OR “multi morbid*”) OR AB(multimorbid* OR “multi morbid*”)
12	TI(“multiple morbid*”) OR AB(“multiple morbid*”)
13	TI(multidisease# OR “multi disease#”) OR AB(multidisease# OR “multi disease#”)
14	TI((concurrent OR cooccur* OR “co occur*” OR multi OR multiple OR “more than one”) N2 (condition# OR disease# OR disorder# OR illness* OR syndrome#)) OR AB((concurrent OR cooccur* OR “co occur*” OR multi OR multiple OR “more than one”) N2 (condition# OR disease# OR disorder# OR illness* OR syndrome#))
15	S9 OR S10 OR S11 OR S12 OR S13 OR S14
16	(MM “Patient Satisfaction+”)
17	TI(patient* N2 (desire# OR experience# OR need# OR opinion# OR prefer* OR priorit* OR satisf* OR value#)) OR AB(patient* N2 (desire# OR experience# OR need# OR opinion# OR prefer* OR priorit* OR satisf* OR value#))
18	TI(patient* N2 (barrier# OR challenge# OR enable* OR facilitat* OR obstacle#)) OR AB(patient* N2 (barrier# OR challenge# OR enable* OR facilitat* OR obstacle#))
19	S16 OR S17 OR S18
20	S8 AND S15 AND S19
